# Improving Outcomes of CT-Guided Malignant Lung Lesion Microwave Ablation by Tract Sealing Using Venous Blood Clot

**DOI:** 10.3390/diagnostics14232631

**Published:** 2024-11-22

**Authors:** Aurimas Mačionis, Gertrūda Maziliauskienė, Rūta Dubeikaitė, Donatas Vajauskas, Dalia Adukauskienė, Irena Nedzelskienė, Marius Žemaitis

**Affiliations:** 1 Department of Radiology, Medical Academy, Lithuanian University of Health Sciences, LT-44307 Kaunas, Lithuania; gertruda.maziliauskiene@gmail.com (G.M.); ruta.pacinskaite@stud.lsmu.lt (R.D.); donatas.vajauskas@kaunoklinikos.lt (D.V.); 2 Department of Intensive Care, Medical Academy, Lithuanian University of Health Sciences, LT-44307 Kaunas, Lithuania; dalia.adukauskiene@kaunoklinikos.lt; 3 Department of Dental and Oral Diseases, Hospital of Lithuanian University of Health Sciences Kauno Klinikos, LT-50161 Kaunas, Lithuania; irena.nedzelskiene@lsmu.lt; 4 Department of Pulmonology, Medical Academy, Lithuanian University of Health Sciences, LT-44307 Kaunas, Lithuania; marius.zemaitis@kaunoklinikos.lt

**Keywords:** lung cancer, lung microwave ablation, pneumothorax, tract sealing, autologous blood clot

## Abstract

**Background:** Complications, particularly pneumothorax, are common following lung interventions and occasionally necessitate further examinations, extend hospital stays, increase treatment costs, and result in long-term health impairment or even death. A few lung intervention tract sealants have been explored to reduce procedure-related complications. **Objectives:** The primary objective of this prospective non-randomized study was to assess the complication rates and risk factors for computed tomography-guided lung microwave ablation (MWA) with autologous blood clot as a tract sealant. **Methods:** Twenty-one patients underwent a total of 26 MWA sessions for lung malignancy followed by injection of the patient’s clotted venous blood into the ablation tract while retracting the coaxial needle. Ablation tract sealing was successful in all MWA sessions. **Results:** Pneumothorax was the only complication observed in five (19.2%) sessions, with one patient (3.8%) requiring chest tube insertion. The male sex was a statistically significant risk factor for pneumothorax (*p* = 0.042), and patients with lung emphysema had almost fivefold higher odds of developing pneumothorax (OR 4.8; 95% CI, 0.617–37.351; *p* = 0.281). **Conclusions:** This study concludes that pneumothorax is the primary complication following lung MWA, and the male sex is a risk factor. Ablation tract sealing with autologous venous blood is a straightforward and inexpensive technique that can reduce the incidence of procedure-related pneumothorax.

## 1. Introduction

There is no denying that the cancer burden is globally increasing. Trachea, bronchus, and lung cancer is currently the most common type of cancer and the leading cause of cancer-related death worldwide, accounting for 2.3 million new cases and 2.0 million deaths in 2021 [1]. Smoking remains the major risk factor for trachea, bronchus, and lung cancer followed by airborne particulate pollution, second-hand smoking, occupational exposure (e.g., asbestos, silica), household air pollution from solid fuels, ionizing radiation, a low-fruit diet, etc. [1,2]. In addition, the lungs are among the most frequent sites for metastatic spread. It is estimated that up to 54% of cancers from different primary sites metastasize to the lungs, most commonly colorectal, breast, renal, and head and neck cancers [3]. An aging population, ongoing exposure to risk factors, and the growing use of imaging techniques and screening programs contribute to the rising number of newly identified primary or metastatic lung lesions.

At present, surgery is considered the treatment of choice for early-stage primary lung cancer and resectable oligometastatic pulmonary disease. Lung thermal ablation techniques (radiofrequency ablation (RFA), microwave ablation (MWA), and cryoablation) are alternative therapeutic approaches that allow direct destruction of tumor lesions by hyperthermal or hypothermal conditions [4]. These procedures are repeatable, minimally invasive, and tissue-preserving because only small incisions are needed to introduce one or a few ablation antennas or probes, and the ablation zone can be adjusted to spare the healthy parenchyma. Regardless of these benefits, thermal ablation is still mostly used in unresectable cases.

The selection of an ablative modality depends on many variables, including the size and location of the tumor, the number of targets, patient comorbidities, and operator experience [4,5]. MWA is a relatively new modality for the treatment of lung tumors. It has several advantages over other thermal ablation techniques, e.g., shorter setup and ablation times, lower sensitivity to the “thermal sink” phenomenon, and the possibility to ablate larger than 3 cm tumors when using several antennas [4]. MWA and RFA are more effective for local disease control than cryoablation [6]. Compared with surgery, MWA can be beneficial due to shorter hospital stays and lower overall medical costs for the treatment [7].

Complications vary among thermal ablation modalities; nonetheless, the safety in terms of major adverse events is comparable between MWA, RFA, and cryoablation [6]. Pneumothorax is the most frequent complication following MWA. Rates for pneumothorax and drainage vary greatly among studies due to the lack of universal patient follow-up protocols for detecting procedure-related complications, the variability in institutional guidelines and general practice methods for managing pneumothorax, and the diversity in study populations regarding potential risk factors. However, recent studies estimate that pneumothorax occurs in 27–52% and requires drainage in 10–24% of lung MWA sessions [8,9]. Other less commonly reported complications following lung MWA include pulmonary hemorrhage, pleural or pericardial effusion, thickening of pericardial layers, pneumonia, lung abscess, and arrhythmia [8,9,10].

Lung biopsy-related complications demand additional examination, prolong hospitalization, and, on average, increase biopsy cost by 4 times [11]. In recent decades, more attention has been drawn to reducing intervention-related adverse events. Several lung biopsy tract sealants have been explored and shown to decrease pneumothorax and/or chest tube insertion rates, such as autologous blood [12,13,14,15], normal saline [16,17], gelatine sponge [18,19], hemostat gelatine powder [20], hydrogel plugs [21,22,23], collagen foam [24], and fibrin glue [25]. To the best of our knowledge, no human studies have been published analyzing tract sealants for lung microwave ablation and only a couple for radiofrequency ablation: using gelatine sponge slurry with an iodinated contrast medium [26] and gelatine torpedoes soaked in an iodinated contrast medium [27].

Autologous blood clot as an MWA tract sealant is an easy-to-use technique requiring no additional expenses and should be investigated in more detail for reducing post-procedure pneumothorax rates.

## 2. Materials and Methods

### 2.1. Study Design

Primary aim: To evaluate the impact of tract sealing using autologous venous blood clot on the rate of pneumothorax following malignant lung lesion microwave ablation.

Secondary aim: To evaluate risk factors for pneumothorax following malignant lung lesion microwave ablation.

This prospective, non-randomized study was approved by the Kaunas Regional Biomedical Research Ethics Committee. Between November 2022 and February 2024, twenty-one patients met the inclusion criteria of this study and underwent MWA for malignant lung lesions.

Inclusion Criteria:

1. Patient Profile:
  - Patients diagnosed with a primary malignant or metastatic lung tumor.
  - Lesions are accessible for microwave ablation at the time of treatment.
  - Maximum diameter of the lesion ≤ 3 cm.
  - The intent of radical treatment.
2. Agreement on curative MWA treatment confirmed by a multidisciplinary team, including an interventional radiologist, radiation therapist, thoracic surgeon, oncologist, and pulmonologist.
3. Medical Suitability:
  - No contraindications for general anesthesia or sedation.
  - No severe coagulopathy or patients must be able and willing to stop antiplatelet medications before the procedure.
4. Consent:
  - Patient agreement to participate in this study provided via a signed informed consent form.

Clinical data and tumor- and procedure-related data were collected to analyze risk factors for pneumothorax and its related intervention. Clinical characteristics included age, sex, presence of lung emphysema, or bullae. The tumor characteristics assessed were tumor histology, the number of lesions, and the size and location of these lesions concerning the pleura. All data were collected during patient consultation prior to the MWA procedure from each patient, their medical records, and imaging studies. After the procedure, the interventional radiologist documented the size of the coaxial needle used, the number of pleural punctures and lesions ablated in the session, the distance of aerated lung transversed, and the duration of ablation. Data obtained from control CT scans included the development and severity of complications and were documented by the operating interventional radiologist alongside the management tactic of observation or chest tube insertion. Delayed post-procedure complications were assessed from the patients and their clinical and imaging records the day following MWA and during subsequent patient consultations.

### 2.2. Microwave Ablation Procedure

Patient preparation included the withdrawal of antiplatelet and anticoagulation medications followed by a fasting period of 8 h before the procedure. Patients underwent sedation or general anesthesia alongside the administration of local anesthetic. The decision on the type of anesthesia was based on the patient’s age, functional status, comorbidities, and expected duration of the procedure.

All procedures were performed by a trained and experienced interventional radiologist under CT guidance using Revolution Ascend 64-slice CT scanner (GE Healthcare, Louisville, KY, USA). A low-dose CT scan protocol (tube voltage 100.0 kV, tube current 50.0–100.0 mA) with a 1.25 mm slice thickness was used for initial, intra-procedural, and follow-up imaging. Based on initial diagnostic imaging, the scanning field was adjusted to visualize the area of the lesion to further minimize radiation exposure. An initial CT scan of the area of interest was obtained to determine the safest and most convenient path for intervention as well as the positioning of the patient. The TATO2 system (Biomedical Srl, approved by 93/42/EEC directive, Florence, Italy) was used in all MWA sessions. The system operating frequency was 2.4–2.483 GHz with a maximum output power of 120 W.

All operations were performed using the coaxial needle technique. A 15 G (gauge) coaxial needle was used to introduce an ablation antenna. Antennas with a diameter of 17 G were used by applying a single puncture or overlapping techniques. The used ablative output power was 30 W.

**Figure 1:**
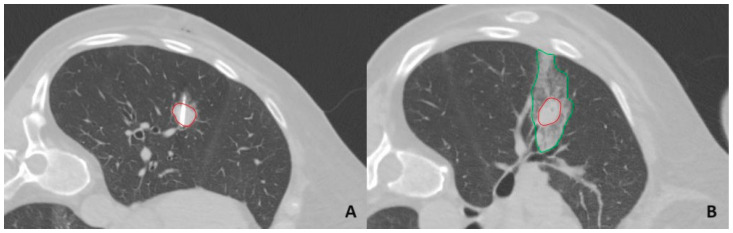
Lung tumor MWA. Image (**A**) shows a tumor transected by the ablation antenna. Image (**B**) shows the post-ablation zone of ground-glass opacity surrounding tumor borders. The red line indicates tumor borders, and the green line indicates ablation zone borders. MWA—microwave ablation.

The ablation procedure was considered successful if the post-ablation area completely encompassed all tumor borders by the end of the procedure (Figure 1).

### 2.3. Tract Sealing Using an Autologous Venous Blood Clot

Before the MWA procedure, each patient had 10–40 mL of their venous blood drawn into an anticoagulant-free syringe. The blood-filled syringe was kept plunger side down for a minimum of 40 min during the procedure. This allows the formed elements to separate from the plasma, facilitating the formation of a blood clot. The plasma was drained prior to tract sealing.

Upon completion of lesion ablation, the microwave antenna was immediately removed, and a syringe with clotted blood was attached to the coaxial needle. Clotted blood was injected while the coaxial needle was withdrawn by 0.5–1 cm at a time, allowing a few minutes between the steps for the clot to firmly set in the ablation tract. The sealing was repeated in the same manner until the coaxial needle was retracted into the chest wall. Typically, 5 to 20 mL of the patient’s venous blood clot was utilized for sealing depending on the number and length of the intervention tracts.

**Figure 2:**
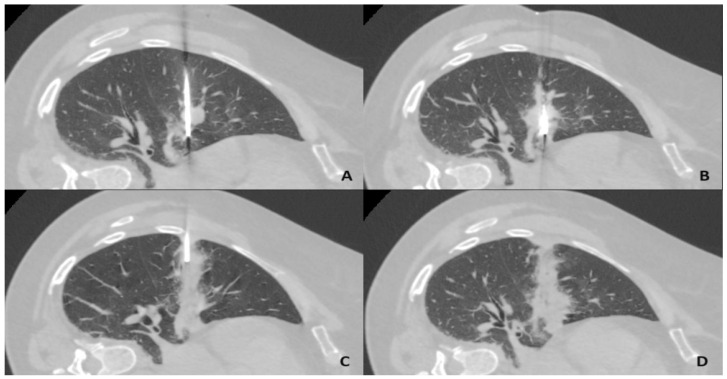
CT-guided lung microwave ablation procedure with tract sealing using clotted autologous blood. (**A**): Right lung middle lobe tumor penetrated by ablation antenna. (**B**): Ablation procedure with the formation of a consolidation zone. (**C**): Ablation tract sealing by injecting autologous blood clot while retracting the coaxial needle. (**D**): Fully sealed ablation tract—focal parenchymal consolidation is present through the entire distance of the transected lung. CT—computed tomography.

The presence of parenchymal consolidation throughout the course of the ablation antenna up to the pleura was a desired finding consistent with successful ablation tract sealing (Figure 2).

Immediately following completion of intervention tract sealing, a sterile patch was applied to the puncture site, and the patient was rolled over to a supine or lateral decubitus position on a puncture-dependent side. If adequate intervention site pressure was not achieved solely through patient positioning, a sandbag was placed at the puncture site to apply additional compression. During recovery, patients were advised to avoid coughing and to remain lying puncture-site down for a minimum of 4 h.

### 2.4. Assessment of Complications

Patient follow-up for MWA-related complications included a first control chest CT scan 10 min after placing the patient on a puncture-dependent side and a second one 24 h following MWA. If the first control scan was deemed abnormal, an additional CT scan was obtained in the following 10–15 min.

Pneumothorax was regarded as significant if it was large upon initial examination; it increased over time; or the patient had experienced breathing difficulty or chest pain on the ipsilateral side or had developed cyanosis, hypotension, or tachycardia. Only pneumothoraces deemed significant were treated with drainage. If a pneumothorax or hemorrhage was considered non-significant during the first control CT scan, it was assessed on an additional follow-up scan. No parenchymal or pleural hemorrhage increasing with time or requiring treatment was observed in this study population.

Pneumothorax was graded in accordance with the Cardiovascular and Interventional Radiological Society of Europe (CIRSE) criteria [28]:

- Grade 1—complication during the procedure that can be solved within the same session; no additional therapy, no post-procedure sequelae, no deviation from the normal post-therapeutic course.
- Grade 2—prolonged observation including overnight stay (as a deviation from the normal post-therapeutic course < 48 h); no additional post-procedure therapy, no post-procedure sequelae.
- Grade 3—additional post-procedure therapy or prolonged hospital stay (>48 h) required; no post-procedure sequelae.
- Grade 4—complication causing a permanent mild sequela (resuming work and independent living).
- Grade 5—complication causing a permanent severe sequela (requiring ongoing assistance in daily life).
- Grade 6—death.

Parenchymal hemorrhage along the sealed intervention tract and minimal post-procedural pleural effusion were not considered complications as these are likely findings consistent with tract embolization using clotted blood.

All individuals were monitored for at least 24 h after the MWA procedure. Those without significant complications on the second control CT scan were discharged on the same day.

### 2.5. Statistical Analysis

Statistical analysis of the data was performed using software packages for the storage and analysis of data, SPSS 29.0 (IBM, Armonk, NY, USA). The Mann–Whitney U test was used to compare the quantitative sizes of two independent samples. The interdependence of qualitative evidence was evaluated using the chi-square (χ^2^) criteria. Depending on the sample size, exact (for a small-sized sample) and asymptomatic criteria were used. Logistic regression analysis was performed to determine the odds ratio predictive value. Differences between groups were considered significant when the level of significance was *p* < 0.05.

### 2.6. Control Group

In our center, we began using autologous blood clots as tract sealants for lung lesion biopsy. Noticing the decrease in post-procedure pneumothorax rates [12] and the technique’s cost-effectiveness and simplicity, we have since implemented it for all lung MWA procedures at our institution. For the purpose of evaluating the effectiveness of autologous blood clot as a tract sealant, we refer to our patients who underwent lung lesion biopsy without tract sealing as the control group [12]. Patient follow-up for the assessment of biopsy-related complications slightly differed from MWA patients and included a control chest CT scan 10 min following patient placement on the biopsy-dependent side and a control chest X-ray 24 h post-procedure. If the control CT scan was deemed abnormal, an additional CT scan was obtained in the following 10–15 min. No significant hemorrhagic complications were observed among the biopsy patients. The criteria for dividing pneumothoraces into clinically significant and insignificant were the same in both studies and are described in detail above. Only significant pneumothoraces following biopsy were drained.

## 3. Results

Twenty-one patients (11 men and 10 women, mean age 67.3 ± 9.3 years; range 48–86 years) were enrolled in this study. A total of 26 MWA sessions were performed in which 30 lesions were ablated. All ablation sessions were successfully completed. Findings consistent with successful ablation tract sealing were observed on control CT scans in all MWA sessions. The histological characteristics of ablated lesions are included in Table 1. Three lesions were repeatedly ablated due to local recurrence observed on follow-up CT scans.

No progressive parenchymal or pleural hemorrhage requiring treatment was noted in this study population. Pneumothorax occurred in five MWA sessions (19.2%), of which one patient had an increasing pneumothorax requiring chest tube insertion (3.8%). The average maximum separation of visceral and parietal pleural surfaces in sessions with pneumothorax was 13.8 mm (range 6–36 mm). The drained patient had a chest tube inserted for three days until pneumothorax was no longer detected on the chest X-ray. No additional intervention was needed, and the patient was discharged four days after MWA with no procedure-related sequelae. Therefore, the complications were graded as Grade 1 or Grade 3 pneumothoraces (Table 2). No procedure-related deaths, events of pneumonia, lung abscess, empyema, pericardial effusion, or arrhythmia was recorded.

In a previous published study conducted in our center, 218 patients underwent lung lesion biopsy, of which for 113 patients, the biopsy tract was sealed using autologous venous blood clot and, for 105 patients, no tract sealing was performed [12]. Comparing the results of the lung lesion biopsy control group with the data from this study, we can observe a decrease in pneumothorax and chest tube insertion rates, though the results are not statistically significant (Table 3).

The analysis of risk factors for pneumothorax following lung lesion MWA is demonstrated in Table 4. Patient- and procedure-related factors were analyzed with respect to the number of MWA sessions, while lesion-related factors were calculated with respect to the number of lesions treated. Among all investigated factors, only the male sex was found to be a statistically significant risk factor for pneumothorax (*p* = 0.042).

Patients with lung emphysema had almost fivefold higher odds of developing pneumothorax (OR 4.8, 95% CI, 0.617–37.351; *p* = 0.281), and lesions in contact with the pleura had almost twice-higher odds of inducing pneumothorax (OR 1.833, 95% CI, 0.150–22.366; *p* = 0.538); however, these findings were not statistically significant. In this study, the only patient who required insertion of a chest tube had emphysema.

## 4. Discussion

Lung microwave ablation has been insufficiently studied so far as a treatment option for lung cancer. Studies comparing the safety and efficacy of thermal ablation methods with more conventional surgical and stereotactic radiotherapy treatment strategies are scarce. Based on existing publications, MWA seems to be safer in terms of clinically significant and life-threatening complications than surgery [10,29]. However, lung ablation procedures typically require CT guidance, and radiation exposure is a considerable factor in patient management. With the widespread implementation of low-dose CT for lung cancer screening, more attention is drawn to limiting radiation exposure during interventional procedures. Several studies have demonstrated a meaningful reduction in the radiation dose with non-inferior diagnostic accuracy and safety with the implementation of low- or ultra-low-dose CT protocols for lung biopsy [30,31]. To lower patient radiation exposure, we performed all the MWA procedures in this study using a low-dose CT protocol while limiting and adjusting the scanning field.

Pneumothorax is the most common adverse event of lung tumor MWA. Some argue that pneumothorax should not be considered a complication as long as it does not prolong hospitalization stay or require specific treatment [32]. However, according to the literature, up to a quarter of lung MWA patients require chest tube insertion due to increasing or clinically significant pneumothoraces. In this study, the rates of pneumothorax and chest tube insertion following tract sealing are lower compared with those in the most recent MWA studies of a larger sample size without tract sealing: 19.2% vs. 27.4–52% for pneumothorax and 3.8% vs. 10.5–23.5% for chest tube insertion [8,9]. To the best of our knowledge, this is the first human study investigating tract sealing following lung MWA. In a rabbit study, tract sealing with gelatine sponge particles reduced the pneumothorax rate from 56.5% to 25.0% (*p* = 0.028) and the aspiration rate from 26.1% to 8.3% (*p* = 0.137) [33].

Autologous blood has been shown to be beneficial for lung lesion biopsy. Topal U and Berkman Y [34] found that bleeding occurring in the intervention tract has a preventative effect on the development of pneumothorax. In support of this, in a different study conducted in our center, we observed that, among patients without lung biopsy tract sealing, those who had parenchymal hemorrhage surrounding the biopsy site were twice less likely to develop pneumothorax [12]. There are some variations in biopsy tract sealing techniques among researchers that use autologous blood. Clayton J et al. [14] and Graffy P et al. [15] used non-clotted blood and were able to significantly lower both the rate of pneumothorax and its related intervention. Malone L et al. [13] used fragmented blood clot and observed a reduction in pneumothorax from 35% to 26% (*p* = 0.12) and chest tube placement from 18% to 9% (*p* = 0.048). In our center, we were able to reduce pneumothorax rates from 31.4% to 21.2% (*p* = 0.087) and chest tube insertion rates from 10.5% to 7.1% (*p* = 0.374) by introducing autologous blood clots into the biopsy tracts [12]. Results of a recent meta-analysis of studies using intraparenchymal injection of autologous blood (both clotted and un-clotted) following lung biopsy have strongly confirmed a significant reduction in both the incidence of pneumothorax and chest tube placement [35]. Results of the subgroup analysis from the study indicate that the effectiveness of tract sealing is improved when clotted blood is used. In agreement with this idea, we continue to believe that clotted blood is more advantageous than non-clotted blood due to its higher density, which should prolong resorption time, thus further reducing the risk of early air leakage.

One would expect a pneumothorax to occur and require drainage more often for lung MWA patients compared with biopsy patients considering the typically larger-diameter needles used and the longer procedure duration for ablation. For instance, in our center, we use 15 G coaxial needles and 17 G ablation antennas for MWA and 16 G coaxial and 18 G biopsy needles for biopsy. Surprisingly, the rates of pneumothorax and chest tube insertion in our center were slightly lower for MWA patients compared with lung lesion biopsy patients with tract sealing: 19.2% vs. 21.2% and 3.8% vs. 7.1%, respectively [12]. These results can, in part, be attributed to the imbalance in the sizes of the study groups: 113 biopsy patients and 26 MWA sessions.

The results of the current study are non-inferior to the ones published by researchers investigating tract sealants for RFA. In Dassa M et al.’s study [26], pneumothorax occurred in 34%, and drainage was needed in 10% of the patients following RFA with tract sealing using gelatine sponge slurry. In Izaaryene J et al.’s study [27] investigating gelatine torpedoes soaked in an iodinated contrast medium as a sealant for RFA, pneumothorax occurred in 47%, and drainage was needed for 8% of the patients.

No meta-analysis of risk factors for pneumothorax following lung MWA can be found in the literature. However, researchers analyzing pneumothorax complicating RFA concluded that male sex, older age, no previous lung surgeries, an increased diameter of aerated lung crossed, and a greater number of tumors treated in a session are risk factors for ablation-related pneumothorax [36]. A few single-center studies established that the duration of the ablation procedure, the size of the ablated lesion, and the presence of invasion of the adjacent lung lobe were associated with an increased incidence of pneumothorax following MWA [37,38]. The male sex and the presence of emphysema were found to be predisposing factors for the insertion of a chest tube to manage pneumothorax [39]. Our results support these statements as we have established that the male sex was a significant risk factor for pneumothorax, and patients with emphysema have higher odds of developing pneumothorax. Due to the small number of patients requiring chest tube insertion, no risk factors for drainage can be evaluated in our study; however, the only patient who was drained was a man and had lung emphysema.

Despite the evident advantages of various tract-sealing materials in reducing pneumothorax rates, the autologous blood technique is exceptional due to the little to no expenses and time resources required for making a blood clot and sealing the tract. Additional steps needed to perform tract sealing are uncomplicated and, therefore, can be quickly mastered. Due to the natural origin of the substance used, no allergic or inflammatory responses should be activated.

There are some limitations to this study. Firstly, all lung tumor MWA sessions performed in our institution were followed by tract sealing. In order to illustrate the efficacy of tract sealing with autologous blood clot, we referred to our previously published data pertaining to lung lesion biopsy as a control group. We acknowledge differences in the procedural lengths, the materials used, and the sizes of the study groups that can influence the results. Secondly, a small MWA patient sample size may be responsible for some of the contradictory trends we observed, e.g., among sessions that resulted in pneumothorax patients being younger, their lesions being smaller, and the duration of ablation and the distance of traversed lung being shorter. Nonetheless, these trends were not statistically significant. Finally, there is a lack of research on this specific topic. The variety of sealants used and differences in MWA techniques, equipment (e.g., the diameter of the antenna and coaxial needle, and the duration of ablation), and the assessment of risk factors and procedure-related complications among institutions pose a challenge for analyzing and comparing our data with other published research.

Despite the need for further prospective control studies to determine the efficacy of this method in more detail and to provide decisive recommendations, we encourage the application of this methodology as routine practice for patients requiring lung biopsy or ablation.

## 5. Conclusions

Pneumothorax is the primary complication following lung MWA. This study identified the male sex as the sole risk factor for pneumothorax after lung MWA. Additionally, patients with lung emphysema and lesions adjacent to the pleura were more likely to experience pneumothorax following the ablation. Lung microwave ablation tract sealing using autologous venous blood clot is a quick, easily applied, and low-cost technique that can reduce the incidence of procedure-related pneumothorax. More detailed controlled trials with larger datasets are needed to accurately determine the role of clotted autologous venous blood in reducing complication rates following lung MWA.

## Figures and Tables

**Table 1:** Histological characteristics of the ablated lesions.

Characteristic	Number of Lesions (%)
Primary lung cancer	18 (60)
Adenocarcinoma (including adenocarcinoma in situ)	15 (50)
Squamous cell carcinoma	3 (10)
Metastatic lung lesions	12 (40)
Hemangioendothelioma	4 (13.3)
Intestinal adenocarcinoma	3 (10)
Hemangiopericytoma	2 (6.7)
Renal carcinoma	2 (6.7)
Melanoma	1 (3.3)
Repeatedly ablated tumors	3
Total	30

**Table 2:** Grading of pneumothorax severity according to the Cardiovascular and Interventional Radiological Society of Europe criteria [28].

Pneumothorax Grade	Number of Patients
Grade 1	4
Grade 2	0
Grade 3	1
Grade 4	0
Grade 5	0
Grade 6	0

**Table 3:** Comparison of complication rates of lung lesion biopsy without tract sealing versus lung microwave ablation with autologous blood clot as a tract sealant. MWA—microwave ablation.

	Complication	Pneumothorax	*p*-Value	Chest Tube Insertion	*p*-Value
Group					
Biopsy without tract sealing		31.4% (33/105)	0.220	10.5% (11/105)	0.458
MWA with tract sealing		19.2% (5/26)		3.8% (1/26)	

**Table 4:** Patient-, procedure-, and lesion-related factors for pneumothorax in patients treated with microwave ablation.

Characteristics			Overall	Pneumothorax	No Pneumothorax	*p*-Value
Number of Sessions			26	5	21	
Patient-related	Age (y)		66.0 ± 8.8	62.6 ± 5.0	66.8 ± 9.4	0.308
	Sex	Men	14	5 (100%)	9 (42.9%)	0.042
		Women	12	0 (0%)	12 (57.1%)	
	Lung emphysema, bullae	Yes	8	3 (60%)	5 (23.8%)	0.281
		No	18	2 (40%)	16 (76.2%)	
Procedure-related	Number of lesions ablated per session	1	22	4 (80%)	18 (85.7%)	1.000
		2	4	1 (20%)	3 (14.3%)	
	Number of pleural punctures	1	20	5 (100%)	15 (71.4%)	0.298
		2	6	0 (0%)	6 (28.6%)	
Number of lesions			30	5	25	
Lesion-related	Contact with pleura	Yes	4	1 (20%)	3 (12%)	0.538
		No	26	4 (80%)	22 (88%)	
	Length of aerated lung traversed (mm)		41.4 ± 12.3	36.6 ± 15.3	42.4 ± 12.0	0.300
	Duration of ablation (min)		20.0 ± 10.2	18.4 ± 7.6	20.4 ± 10.8	0.829
	Lesion’s maximum diameter (mm)		14.0 ± 6.6	11.4 ± 6.8	14.5 ± 6.6	0.327

## Data Availability

The raw data supporting the conclusions of this article will be made available by the authors on request.
